# Suicide attempts among students of higher education, Nelson Mandela Bay Municipality, South Africa

**DOI:** 10.4102/safp.v64i1.5609

**Published:** 2022-11-08

**Authors:** Adeyinka A. Alabi

**Affiliations:** 1 Department of Family Medicine, Faculty of Health Science, Walter Sisulu University, Port Elizabeth, South Africa; 2 Department of Family Medicine, Dora Nginza Provincial Hospital, Port Elizabeth, South Africa

**Keywords:** suicide attempts, college students, risk factors, suicide, prevalence

## Abstract

**Background:**

Worldwide, death by suicide is a leading cause of death among young people, and students of higher educational institutions constitute a vulnerable group. This study aimed to determine the lifetime prevalence and associated factors of suicide attempt among students of a higher education institution in Nelson Mandela Municipality.

**Methods:**

A cross sectional study was conducted among students of East Cape Midland College in Nelson Mandela Municipality. The participants were selected by stratified random sampling and a standardised self-administered questionnaire was used to collect data.

**Results:**

The prevalence of lifetime suicide attempts was 16.0% among the participants. Multivariate logistic regression analysis revealed higher odds of suicide attempts among participants who: experienced bullying (OR: 1.66, CI: 1.05–2.61; *p* < 0.001), had underlying medical conditions (OR: 3.27, CI: 2.08–5.14; *p* < 0.001), had abnormal body weight perceptions (OR: 1.64, CI: 1.03–2.62; *p* < 0.05), had experienced sexual abuse (OR: 5.72, CI: 2.86–11.45; *p* < 0.001), or had someone very close who had experienced sexual abuse (OR: 1.77, CI: 1.02–3.05; *p* < 0.05).

**Conclusion:**

This study identified history of sexual abuse, bullying, perceptions of abnormal body weight and underlying medical conditions as associated risk factors of suicide attempts among the participants. The high prevalence of suicide attempts among the participants (16%) demonstrates the urgent need for campus-based interventions and prevention strategies aimed at addressing the identified associated factors.

## Introduction

Suicide is a significant public health problem globally, with over 700 000 deaths from suicide annually and many more people attempting suicide.^1^ It also constitutes the fourth leading cause of death among adolescents and young people aged 15–29 years, among whom students of higher education institutions (HEIs) represent a significant subset.^2^ This age group (15–29 years) also represents a period of high vulnerability to mental illness, which is often undiagnosed and untreated.^3^

The estimated suicide rate in South Africa is 23.5 per 100 000, which translates to about 14 000 deaths from suicide per annum.^1^ It is the second leading cause of death among young people aged 15–29 in the country.^4^ The years of productive life lost and the economic costs of suicide are a burden to both families and the nation.^5^

Globally, the morbidity from suicide attempts among young people is also significant, with a reported 20.5% of the total population of young people attempting suicide in low- and middle-income countries.^6^ A systematic review of studies from 18 sub-Saharan countries reported a prevalence of 16.9% among young people in the region.^7^ There is no national data on the rate of suicide attempts (as opposed to suicides) in South Africa; however, it has been estimated to be approximately 280 000 per annum, based on a figure of 20 attempts for every suicide death.^1^ The use of health facilities in the immediate post-attempted suicide period by patients constitutes a great problem to the already overburdened public health sector. In this study, suicide attempt is defined as a non-fatal self-directed injurious behaviour with the intent of ending one’s life.^8^

Young people at HEIs face significant stress in the form of academic workloads, poor funding, peer pressure and separation from family.^9,10^ These stressors increase their vulnerability to various mental disorders such as suicidal behaviour, depression, anxiety and substance abuse.^11^ The prevalence of suicide attempts reported in the literature varies from region to region and may depend on the terminology used, the sampled population, sample size and study design.^12^ In a survey conducted among first-year university students in South Africa, Bantjes, Breet, Saal et al. (2019) reported a 8.6% prevalence rate of suicide attempts%.^12^ Another local study among students of 16 high schools noted a prevalence rate of 14.8%.^13^ This variation in prevalence could be because of differences in the sampled population, with the former study conducted among university students and the latter among high school learners, who might have been less well equipped to handle pressures than their older counterparts. Suicide prevention strategies should be aimed at offering context-specific interventions to mitigate the risk factors among vulnerable groups. Some of the risk factors for suicide attempts in the two studies listed previously were: mental illness, family conflict, romantic relationship crises, loneliness, bullying, lower socioeconomic status, adverse childhood events, sexual and physical abuse and academic failure.^7,14^ There is a dearth of studies on the prevalence of and factors associated with suicide attempts among students of technical and vocational education and training (TVET) colleges in South Africa, a gap which this study aimed to fill.

## Methods

### Setting

The study was planned to take place in East Cape Midland College (EMC) and Nelson Mandela University in the Eastern Cape, South Africa. However, the researcher was able to survey only EMC before the South African government declared a state of national disaster to curtail the spread of COVID-19 pandemic on 15 March 2020. East Cape Midland College is a TVET college that offers courses in engineering, business and occupational training. It has seven campuses spread across Uitenhage in Nelson Mandela Municipality. It is a non-residential institution and therefore all students reside off-campus. During the period of the study, a total of 8563 students were registered in all campuses of EMC and were eligible to participate in the study.

### Study design and duration

An institution-based cross-sectional study was conducted between 15 January 2020 and 15 March 2020.

### Sample size calculation

The sample size was calculated based on the formula:

$$n={\text{Z}}^{2}\text{p}(1-\text{p})/{\text{e}}^{2},$$ [Eqn 1]

where *n* = sample size, *Z* = desired confidence level, *p* = prevalence of suicide attempts and *e* = margin of error.

The researcher worked on the assumption of a 17.0% prevalence rate of suicide attempts among young people,^6^ with a margin of error set as 2.5% and a confidence interval of 95%. An initial sample of 868 participants was calculated. An additional 34 were added in anticipation of incomplete responses to give a total of 902 sample size.

To be included in the study, participants had to be: (1) at least 18 years old, (2) registered for the 2019/2020 academic year and (3) willing to sign the consent form and complete a 15-min questionnaire. Participants were excluded if they: (1) did not complete the questionnaire or (2) declined to sign the consent form.

### Sampling technique

A three-stage stratified random sampling technique was used to select the participants. In the first stage, four campuses were randomly selected from the seven campuses. In the second stage, the lecture rooms in each of the campuses were clustered into groups based on department and the year of study of potential participants. During the third stage, a random selection of lectures rooms from the clusters was conducted and all students who were present in the selected classes were recruited into the study voluntarily. Questionnaires were distributed by research assistants after participants had signed consent forms.

### Questionnaire development

The researcher designed a questionnaire based on an extensive review of the literature. The dependent and independent variables of interest are displayed in Table 1.

**TABLE 1:** Dependent and independent variables of suicide attempts.

Variable	Measurement	Variable description	Example of measurement item
Suicide attempts	Lifetime suicide attempt	Binary	In your lifetime, have you ever attempted suicide? Response: Yes or No.
	Number of attempts	Binary	How many times have you attempted suicide? Response: Once or more than once.
Health service utilisation following suicide attempt		Binary	Did you use any of the following health services? (1) mental health services, (2) local clinic, (3) casualty at hospital, (4) in-patient care, (5) intensive care unit and (6) did not seek any healthcare. Response: Yes or No.
Method of suicide attempt	Method of suicide attempt	Score	Assessed by asking the participant to choose the method used from any of the following: drug overdose, cutting of body part, hanging, jumping from height, shooting or other.
Socioeconomic status		Score	Economic status was assessed by obtaining information about the employment status of parents, total monthly family income and number of household members. Participants were also asked to rate their perceptions of their financial support, from very poor to very good.
Chronic illnesses	Assessment of chronic medical and psychiatric illness	Binary	Have you ever been diagnosed by a health professional with any of the following chronic illnesses: chronic pain, HIV, depression, anxiety, bipolar disorder, schizophrenia or unknown chronic illness? Responses: coded as 1 for ‘No’ and 2 for ‘Yes’.
Substance use	Alcohol use	Binary	Do you drink an alcoholic drink? Responses: code as 1 for ‘No’ and 2 for ‘Yes’
	Cannabis use	Binary	Were you previously drinking alcohol? Responses: code as 1 for ‘No’ and 2
Academic status	Experienced academic failure	Binary	Have you ever experienced an academic failure? Response: Yes or No.
Experience of bulling		Binary	Have you ever been bullied by another student? Response: Yes or No.
Sexual abuse	Self	Binary	Have you ever been forced to have sex? Response: Yes or No.
	Someone very close	Binary	Has someone very close to you ever been forced to have sex? Response: Yes or No.
Conflict in relationship		Binary	Has your partner ever insulted or beaten you in the past? Responses: coded as 1 for ‘No’ and 2 for ‘Yes’ Have you had a failed relationship? Responses: coded as 1 for ‘No’ and 2 for ‘Yes’
Body weight perceptions		Binary	Participants’ perception of their body weight was assessed by asking, ‘How do you feel about your physical appearance?’ Options were ‘I feel perfect’, ‘moderately good’ or ‘bad’.

Pilot testing of the questionnaire was conducted with 10 students from one of the campuses. Participant feedback was used to make minor adjustment to the questionnaire. The results of the pilot study were excluded from the primary study.

### Data analysis

Descriptive analysis was used to describe the socio-demographic characteristics of the participants, with the variables reported as means, frequencies and percentages. A chi-square test and bivariate analysis were employed to assess associations between suicide attempts and the independent variables. Multiple logistic regression analysis was performed to determine the factors independently associated with suicide attempts among the participants after adjusting for confounding variables. The associations were measured using odds ratios (OR) and 95% confidence intervals (95% CI), with the significant level set at *p* < 0.05. All data were analysed using STATA software, version 15.0 (Stata Corporation, College Station, TX, US).

### Ethical considerations

The Ethics Committee of Walter Sisulu University approved the study protocol and issued ethical clearance for the study (Reference: 038/2019). Permission was also obtained from the Department of Health (Reference: EC_201912005). The selected participants were provided with information leaflets explaining the objective and processes of the study. All participants provided written consent before the commencement of the study. There was no financial inducement to participate. The right to privacy and confidentiality of information was respected during and after the study. Anonymity was ensued during the study, with no participant identifiers collected. Permission was also obtained from the campus manager of each of the campuses before distribution of the questionnaires.

## Results

### Descriptive findings

A response rate of 94.8% was achieved in this study, with 855 returned questionnaires out of 902 recruited into the study. Missing information warranted the exclusion of 29 questionnaires from the final analysis. A total of 826 samples were included in the final analysis: 527 women (61.6%) and 317 men (38.4%).

The mean age of the participants was 20.49 (standard deviation [s.d.]:1.88) and ranged from 18 to 24 years. Among the participants, 527 were female (61.6%), 582 (70.6%) were from an urban area and the majority, 382 (44.7%), were in their 1st year of study. The distribution of participants’ family structures were single-parent families (44.8%), two-parent families (42.7%) and foster parents (12.5%). The single-parent family structure was more common among female participants than among male participants (47.2% vs 41%). Approximately one quarter of the participants felt that their financial needs were met. About three-quarters of the participants reported alcohol usage and one-fourth reported having smoked cannabis. Detailed demographic characteristics of the participants are depicted in Table 2.

**TABLE 2:** Demographic characteristics of participants.

Variables	Male		Female		All participants	
	*n*	%	*n*	%	*n*	%
All respondents	317	38.4	509	61.6	826	100.0
**Family structure**						
Single parent	130	41.0	240	47.2	370	44.7
Both parents	149	47.0	204	40.1	353	42.7
Foster parents	38	12.0	65	12.8	103	12.5
**Parents occupation**						
Both employed	68	21.5	81	15.9	149	18.0
One employed	124	39.1	220	43.2	344	41.6
Non-employed	125	39.4	208	40.9	333	40.3
**Place of residence**						
Rural	102	32.2	142	27.9	244	29.5
Urban	215	67.8	367	72.1	582	70.5
**Rate family financial support**						
Very poor	21	6.6	48	9.4	69	8.4
Poor	44	13.9	98	19.3	142	17.2
Moderate	140	44.2	182	35.8	322	39.0
Good	77	24.3	114	22.4	191	23.1
Very good	35	11.0	67	13.2	102	12.3
**Have all needs met**						
Yes	144	45.4	227	44.6	371	44.9
No	173	54.6	282	55.4	455	55.1
**Alcohol use**						
Currently use alcohol	164	51.7	234	46.0	398	48.2
Previously used alcohol	74	23.3	142	27.9	216	26.2
I never used alcohol	79	24.9	133	26.1	212	25.7
**Use cannabis**						
Yes	93	29.3	79	15.5	172	20.8
No	224	70.7	430	84.5	654	79.2
**Have underlying medical conditions**						
Yes	66	20.8	139	27.3	205	24.8
No	251	79.2	370	72.7	621	75.2
**Experienced education failures**						
Yes	106	39.8	160	31.4	266	32.2
No	211	66.6	349	68.6	560	67.8
**Experienced sexual abuse**						
I am a survivor	11	3.5	42	8.3	53	6.4
Someone close is a survivor	27	8.5	98	19.3	125	15.1
I have never experienced sexual abuse	279	88.0	369	56.9	648	78.5
**Experienced unintended pregnancy**						
Yes	24	7.6	57	11.2	81	9.8
No	293	92.4	452	88.8	45	90.2
**Conflictual relationship**						
Yes	69	21.8	141	27.7	210	25.4
No	248	78.2	368	72.3	616	74.6
**Body weight perceptions**						
Normal	246	77.6	367	71.1	613	74.2
Abnormal	71	22.4	142	27.9	213	25.8
**Feel your life is better off than friends**						
Yes	150	47.3	202	39.7	352	42.6
No	167	52.7	307	60.3	474	57.4

### Prevalence of suicide attempts

The overall reported lifetime prevalence of suicide attempts among participants was 16.0%. The prevalence of suicide attempts was higher among female participants (21.6%) compared to male participants (6.6%). Far more participants had made multiple suicide attempts than those who had made a single attempt (62.1% vs 37.9%).

### Factors associated with suicide attempts

As shown in Table 3, in a crude multiple logistic model analysis, male participants feeling better off financially than their friends and having all their needs met were associated with lower odds of suicide attempts. After adjusting for confounding variables, only male sex was a significant variable.

**TABLE 3:** Multivariable model showing factors associated with suicide attempts.

Variable	Made suicide attempts		Unadjusted odds ratio		Adjusted odds ratio	
	*n*	%	*n*	%	*n*	%
All	132	16.0	-	-	-	-
**Age**						
16–19	41	15.2	0.92	0.61–1.37	1.06	0.65–1.73
20–24	91	16.4	1	-	1	-
**Sex**						
Male	22	6.6	0.27**	0.17–0.44	0.32**	0.19–0.55
Female	110	21.6	1	-	1	-
**Experienced bullying**						
Yes	76	24.8	2.74**	1.87–4.00	1.66*	1.05–2.61
No	56	10.8	1	-	1	-
**E xperienced academic failure**						
Yes	39	14.7	0.86	0.58–1.30	0.74	0.46–1.19
No	93	16.6	1	-	1	-
**Feel better off than friends**						
Yes	44	12.5	0.63*	0.42–0.93	1.11	0.69–1.79
No	88	18.6	1	-	1	-
**Have underlying medical conditions**						
Yes	70	34.1	4.68**	3.17–6.91	3.27**	2.08-5.14
No	62	10.0	1	-	1	-
**Relationship conflicts**						
Yes	52	24.8	2.21**	1.49–3.26	1.48	0.93–2.37
No	80	13.0	1	-	1	-
**Bodyweight perception**						
Not normal	58	27.2	2.73**	1.85–4.02	1.64*	1.03–2.62
Normal	74	12.1	1	-	1	-
**Feeling about body appearance**						
Perfect	40	10.9	0.37*	0.20–0.66	0.68	0.33–1.41
Moderate	71	19.0	0.70	0.40–1.23	0.83	0.42–1.63
Bad	21	25.0	1	-	1	-
**All needs met**						
Yes	44	11.9	0.56*	0.38–0.83	0.72	0.44–1.17
No	88	19.4	1	-	1	-
**Family financial support**						
Poor	47	22.3	1.92*	1.20–3.08	1.18	0.65–2.14
Moderate	47	14.6	1.15	0.72–1.82	0.98	0.58–1.68
Good	38	13.0	1	-	1	-
**Experienced sexual abuse**						
I am a survivor	29	54.7	10.31**	5.68–18.71	5.72**	2.86–11.45
Someone very close is	35	28.0	3.32**	2.09–5.28	1.77*	1.02–3.05
Never experienced sexual abuse	68	10.5	1	-	1	-
**Alcohol use**						
Currently drink alcohol	75	18.8	2.66*	1.53–4.64	1.63	0.87–3.04
Previously drank alcohol	40	18.5	2.61*	1.43–4.76	1.68	0.86–3.27
Never used alcohol	17	8.0	1	-	1	-
**Cannabis use**						
Yes	39	22.7	1.77*	1.16–2.67	1.41	0.82–2.42
No	93	14.2	1	-	1	-

* , *p* ≤ 0.001;

** , *p* ≤ 0.001.

Also, the odds for suicide attempt were higher among participants who had experienced bullying, had underlying medical conditions, had relationship conflicts, had poor family financial support, currently drank alcohol, previously drank alcohol, used cannabis, had abnormal body weight perception, had abnormal body image feeling or had an experience of sexual abuse or a person close to them who had experienced sexual abuse.

The relationship between chronic medical conditions (Table 4) and suicide attempts was also explored. However, no reliable inference between individual chronic illness and suicide attempts owing to the low prevalence of the medical illnesses in the sample could be made.

**TABLE 4:** The relationship between chronic medical conditions and suicide attempts

Medical condition	Attempted suicide		Never attempted suicide		*p*
	*n*	%	*n*	%	
**Have chronic pain**					
Yes	23	25.6	67	74.4	0.009
No	109	14.6	627	85.2	-
**HIV positive**					
Yes	13	44.8	16	55.2	< 0.001
No	119	14.9	678	85.1	-
**Have had depression**					
Yes	39	42.9	52	57.1	< 0.001
No	93	12.7	642	87.3	-
**Anxiety**					
Yes	14	26.4	39	73.6	0.031
No	118	15.3	655	84.7	-
**Bipolar**					
Yes	7	46.7	8	53.3	0.005
No	125	15.4	686	84.6	-
**Schizophrenia**					
Yes	0	0.0	2	100.0	0.706
No	132	16.0	692	84.0	-
**Unknown medical condition**					
Yes	5	29.4	12	70.6	0.120
No	127	15.7	682	84.3	-

HIV, Human immunodeficiency virus.

The relationship between the total number of people with associated factors for suicide and suicide attempts was evaluated in multivariable analysis. After controlling for confounding factors in multiple logistic analysis, only the following factors were statistically significant: experienced bullying (OR: 1.66, CI: 1.05–2.61; *p* < 0.001), had underlying medical conditions (OR: 3.27, CI: 2.08–5.14; *p* < 0.001), had abnormal body weight perception (OR: 1.64, CI: 1.03–2.62; *p* < 0.05), experienced sexual abuse (OR: 5.72, CI: 2.86–11.45; *p* < 0.001), had someone very close who had experienced sexual abuse (OR: 1.77, CI: 1.02–3.05; *p* < 0.05).

### Methods of suicide attempts among the participants

Drug overdose was the commonest method of suicide attempt reported by the participants.

The methods of suicide attempts reported by the participants are shown in Table 5.

**TABLE 5:** Methods of suicide attempt.

Method	Number	Percentage
Drug overdose	79	59.8
Cutting hand with sharp objects	32	24.2
Hanging	13	9.8
Jumping from height	5	3.8
Shooting	1	0.8

## Discussion

The results of this study may not be comparable with findings from previous similar studies of higher educational institutions because of the peculiarity of the student population of TVET colleges. These colleges were created to capacitate students with practical skills; they accept students who have passed Grades 9, 10, 11 or 12. Thus, it is appropriate to compare the findings of our study with the results of similar studies in high schools. This study revealed a high prevalence of lifetime suicide attempts, at 16.0%, among students at the college. The finding is consistent with the 16.9% finding in a systematic review from 18 sub-Saharan countries.^7^ It is also in line with the 17.0% reported in a meta-analysis of studies from 59 low- and middle-income countries.^6^ It cannot be said with certainty whether poor differentiation of self-harm from suicide attempts by the responders played a role in the high prevalence uncovered. Fairly similar results of 15.6 %, 16.2% and 15.5% were documented among adolescents and young people in studies from Benin, Ethiopia and Eswatini respectively.^15^ However, the finding in this study is higher than the 8.9% value reported among US high school students.^16^ The finding of this study is also higher than the 8.6% reported in a previous South Africa study.^12^ The differences in the prevalence of suicidal attempts in various studies could be attributed to differences in the prevailing economic climate. Moreover, the absence of a psychologist and other mental health services at the college means that there was no help available on campus to assist students going through psychological strain. Financial hardship coupled with the non-availability of mental health services at the time of the study might have contributed to the high prevalence of suicide attempts reported. The later period of data collection was hampered by the closure of institutions of higher learning as a measure to reduce the spread of COVID-19. The general aura of hopelessness and despair that characterised the global spread of COVID-19 and the preparation programme to curtail the outbreak might also have contributed to the high prevalence of suicide attempts in this study.^17^

However, the prevalence of suicide attempts in this study is lower than the 22.2%, 18.5% and 23.2% reported in school-based surveys in Ghana, Mozambique and Benin respectively.^18,19,20^ The higher prevalences may be attributed to higher proportion of participants: younger than 18 years; and anxiety and bullying experienced in the previous studies.

The study also found that suicide attempts were higher among females than males, which is consistent with what has been reported in the literature.^13,18^ This higher rate may be attributed to various factors, such as a higher incidence of: major depressive disorder,^11^ bullying victimisation^21^ and sexual assault,^22^ all of which have been reported in previous studies also. In contrast, a study in Ethiopia reported that more male participants attempted suicide than female participants.^19^

More than one-quarter of the participants reported having attempted suicide on multiple occasions. Previous studies have shown that suicide attempts are a predictor of future suicide attempts and suicide deaths.^23,24^ Therefore, an encounter with a person who has previously attempted suicide presents an opportunity to screen for risk factors and offer appropriate intervention to ameliorate the risk of further attempts.

Among those who had attempted suicide, drug overdose (60.0%) was the commonest method used, followed by cutting (24.0%). This is consistent with the findings of other studies.^25^ Drug overdose fatalities depend on the type and amount of substance taken. The identification of commonly ingested drugs with fatal outcomes should prompt the implementation of measures to limit access to such medication. Furthermore, local health centres and district hospitals should be regularly updated on the managements of drug overdose cases.

Factors negatively associated with suicide attempts were bullying-related victimisation, chronic medical conditions and sexual assault.

### Bullying-related victimisation

Findings from this study show that the likelihood of suicide attempts was higher among participants who had experienced bullying. This finding is consistent with the results of previous studies that have reported a strong association between a history of being bullied and suicide attempts.^18^ The high rate of suicidal behaviour among victims of bullying may be attributed to loneliness, a perception of being unwanted by peers and a thwarted sense of belonging.^26^ This study noted that 76 (24.8%) students had experienced bullying and had attempted suicide. The association between being bullied and suicidal behaviour highlights the urgent need to introduce interventions to reduce bullying-related victimisation of young people.

### Chronic medical conditions

In this study, there was a low reported rate of mental illness, with 10.0%, 5.9%, 1.7% and 0.2% of participants reporting experiences of depression, anxiety, bipolar disorder and schizophrenia, respectively. Although participants were assured of privacy and confidentiality in this study, it is possible that fear of stigmatisation informed this low reported incidence of mental illness. Non-disclosure of HIV status owing to stigmatisation could also explain the low prevalence of HIV (3.3%) reported among the participants. The study found that the chances of suicide attempts were higher among participants who had chronic medical conditions than among those who did not. This finding is in line with results from other studies that show a strong link between mental illness and suicide attempts.^27,28^ Antipsychotics, mood stabilisers, antidepressants and psychotherapy have been demonstrated to reduce the suicide rate among mental illness sufferers.^29,30^ Instituting campus-based health screening that allows early detection and initiation of effective treatment among college students should be one of the preventative measures taken to reduce suicide attempts among students.

### Sexual assault

The likelihood of suicide attempt was higher among participants who had a personal history of sexual assault or who were close to someone who had experienced sexual assault. This finding is in line with findings of other studies that reveal a consistent relationship between early sexual assault and increased risk of suicide attempts.^31^ The lifetime prevalence of sexual assaults reported among adolescents and university students in South Africa is high, at 37.9%.^32^ Calls for urgent interventions to reduce the incidence of sexual assaults among college students should be heeded, while those who are already victims should be properly supported and rehabilitated.

### Abnormal body weight perception

Abnormal body weight perception was significantly associated with likelihood of suicide attempt among the participant (OR: 1.64, CI: 1.03–2.62). This result is consistent with the findings from similar studies that reported a higher odd of suicide attempts among participants with abnormal perception of their body weight.^33,34^ People view normality of body weight based on media portrayal of thinness as the standard of attractiveness and likeness.^35^ The pressure from trying to actualise this idealised body weight by the adolescents and young people increased their vulnerability to low self-esteem, suicidal behaviour and other mental illness.^35,36^ College staff screening for abnormal body weight perception and other risk factors of suicide attempts can be utilised to identify students that need further assessment and intervention.

## Strength

According to the author’s knowledge, this is the first published article study to report factors associated with suicide attempts among students of higher institution of learning in the Eastern Cape province, South Africa. Furthermore, the utilisation of a multi-stage cluster random sampling ensured that representative sample were recruited for the study. The findings of this study therefore provide a reliable database for further studies that will cut across.

## Limitations

Firstly, the study is limited by the cross-sectional design which shows only associations between various factors and suicide attempts. The self-reported nature of the data might also have been a limitation, by introducing an element of bias. The investigator limits the extent of social desirability bias by anonymising the data collection.

Secondly, the study took place at only one higher educational institution in Nelson Mandela Municipality; therefore, the findings of the study may not be generalised to other higher educational institutions.

Lastly, the study did not explore the association between culture factors and suicide attempts, whether the cultural views of the participants affected their disclosure of suicide attempts is not known with certainty in this study.

## Conclusion and recommendations

Suicide attempts were common among the participants, at 16.0%. Associated factors were bullying, an experience of sexual abuse, whether in the participant or in someone close to the participant, a chronic medical condition and negative perception of body weight. The findings of this study highlight the urgent need for the college to put in place suicide prevention interventions on campus. Furthermore, it also highlights the need for programmes that address the mental health needs of all students. A campus-based wellness clinic that screens and manages the identified risk factors should be given consideration by the stakeholders of the college and by other institutions of higher learning.
